# A nonparametric regression-based linkage scan of rheumatoid factor-IgM using sib-pair squared sums and differences

**DOI:** 10.1186/1753-6561-1-s1-s99

**Published:** 2007-12-18

**Authors:** Saurabh Ghosh, P Samba Siva Rao, Gourab De, Partha P Majumder

**Affiliations:** 1 Human Genetics Unit, Indian Statistical Institute, 203 B.T. Road, Kolkata 700 108, India

## Abstract

Parametric linkage methods for quantitative trait locus mapping require explicit specification of the probability model of the quantitative trait and hence can lead to misleading linkage inferences when the model assumptions are not valid. Ghosh and Majumder developed a nonparametric regression method based on kernel-smoothing for linkage mapping of quantitative trait locus using squared differences in trait values of independent sib pairs, which is relatively more robust than parametric methods with respect to violations in distributional assumptions. In this study, we modify the above mentioned nonparametric regression method by considering local linear polynomials instead of the Nadaraya-Watson estimator and squared sums of sib-pair trait values in addition to squared differences to perform a genome-wide scan of rheumatoid factor-IgM levels on sib pairs in the Genetic Analysis Workshop 15 simulated data set. We obtain significant evidence of linkage very close to the quantitative trait locus controlling for RF-IgM. We find that the simultaneous use of squared differences and squared sums increases the power to detect linkage compared to using only squared differences. However, because of all the sib pairs are selected for rheumatoid arthritis, there is reduced variance of RF-IgM values, and empirical power to detect linkage is not very high. We also compare the performance of our method with two linear regression approaches: the classical Haseman-Elston method using squared sib-pair trait differences and its extension proposed by Elston et al. using mean-corrected sib-pair cross-products. We find that the proposed nonparametric method yields more power than the linear regression approaches.

## Background

Heritable quantitative characters are precursors of a clinical end-point trait. Because end-point traits are usually binary in nature (affected/unaffected) and hence contain minimal information on variation within trait genotypes, it may be statistically more powerful to use a correlated quantitative phenotype for identifying genes for the underlying complex trait. Unlike qualitative or binary traits, which can be characterized completely by allele frequencies and genotypic penetrances, quantitative traits require a stronger layer of modeling: the probability distribution of the underlying trait. Thus, compared to likelihood-based approaches like variance components [1,2], which require explicit specification of the probability distribution of the quantitative trait, nonparametric methods for quanitative trait loci (QTL) mapping are more robust to deviations in distributional assumptions. Ghosh and Majumder [3] developed a nonparametric regression method based on Nadaraya-Watson kernel-smoothing [4,5] for linkage mapping of QTLs using squared differences in quantitative trait values of independent sib pairs. However, studies have shown that information on linkage can be increased by using squared sib pair sums in addition to squared differences [6,7]. Moreover, local linear polynomials provide better nonparametric regression fits [8,9] compared to the Nadarya-Watson estimator. In this study, we modify the nonparametric regression method of Ghosh and Majumder to incorporate squared sums in conjunction with squared differences and use local linear polynomials instead of the Nadaraya-Watson estimator to perform a genome-wide linkage scan of rheumatoid factor (RF)-IgM, a quantitative phenotype correlated with rheumatoid arthritis affection status in the simulated data of Genetic Analysis Workshop 15 (GAW15). We evaluate the gain in power by using squared sib-pair sums in addition to squared differences. We also compare the performance of our nonparametric method with the classical Haseman-Elston linear regression method [10] using sib-pair squared differences as well as its extension proposed by Elston et al. [7] using sib-pair mean-corrected cross-products, which can be expressed as a linear combination of squared differences and mean corrected squared sums.

## Methods

### Data description

For our analyses, we used data on RF-IgM levels and genome-wide information on 730 microsatellite marker loci distributed over the 22 autosomal chromosomes. Our method utilizes marker genotype data on 1500 independent sib pairs and their parents for identity-by-descent (IBD) computations. The nonparametric regressions for the linkage scan are based on the RF-IgM and IBD data. We performed our analyses on all 100 available replicates.

### Statistical methodology

Suppose *y*_*ij *_denotes the RF-IgM of the *j*^th ^sib in the *i*^th ^family, *i *= 1, 2,..., 1500; *j *= 1, 2; and ${\hat{\pi }}_{ip}$ denotes the estimated IBD score for the *i*^th ^sib pair at an arbitrary point *p *on the genome. We define *U*_*i *_= (*y*_*i*1 _- *y*_*i*2_)^2^ and *V*_*i *_= (*y*_*i*1 _+ *y*_*i*2_)^2^. The classical Haseman-Elston method [10] and its extensions [6,7], which involve a linear regression of squared differences (or suitable alternative functions) of sib-pair trait values (*U*_*i *_values) on estimated marker IBD scores (${\hat{\pi }}_{ip}$ values) are adversely affected by the increase in dominance at the QTL. Thus, a more robust strategy is to estimate empirically the nature of the functional relationship between the two variables.

Following Ghosh and Majumder [3], we assume a nonparametric regression model:

$$U_{i}=\psi ({\hat{\pi }}_{ip})+e_{i};i=1,2,...,1500,$$

where *ψ *is a real valued function and *e*_*i *_values are random errors. The functional form of *ψ *is estimated using a kernel smoothing technique [6] with kernel function:

*k*(*x*) = 3/4(1 - *x*^2^), |*x*| < 1;

0, otherwise.

Ghosh and Majumder [3] had used the Nadaraya-Watson estimator for the prediction of *U*_*i *_values. There is now increasing evidence that local polynomials have lower prediction errors [6,7] than the Nadaraya-Watson estimator. We used a local linear polynomial to predict *U*_*i *_as follows: $U_{i}=\hat{\psi }({\hat{\pi }}_{ip})=\sum_{j}\kappa (\frac{{\hat{\pi }}_{ip}-{\hat{\pi }}_{jp}}{h})\{\beta_{0}+\beta_{1}{\hat{\pi }}_{jp}\}/\sum_{j}\kappa (\frac{{\hat{\pi }}_{ip}-{\hat{\pi }}_{jp}}{h}),$,

where *h *is the "optimal" window length in the kernel smoothing procedure obtained using "leave-one-out" cross-validation; and *β*_0 _and *β*_1 _are the weighted least squares estimators of the local linear regression of *U*_*j *_on ${\hat{\pi }}_{jp}$ with weights as

$$\kappa (\frac{{\hat{\pi }}_{ip}-{\hat{\pi }}_{jp}}{h})/\sum_{i}\kappa (\frac{{\hat{\pi }}_{ip}-{\hat{\pi }}_{jp}}{h}).$$

To assess the significance of our regression, we used a diagnostic measure [11]$\Delta =1-\sum_{\iota =1}^{1500}{\left\{U_{i}-\hat{\psi }({\hat{\pi }}_{ip})\right\}}^{2}/\sum_{\iota =1}^{1500}{\left(U_{i}-\bar{U}\right)}^{2}$. We note that the proposed measure Δ is an analog of *R*^2^, the square of the correlation coefficient between the response variable and the explanatory variable, which is used in linear regression as a measure of the proportion of variance of the response variable explained by the explanatory variable. One can evaluate the significance of the observed Δ empirically by generating random IBD scores under the null hypothesis of no linkage, while preserving the actual RF-IgM values.

There have been suggestions that using squared differences in conjunction with squared sums of sib-pair trait values may be a more powerful linkage strategy compared to using squared differences only [6,7]. In order to explore this hypothesis, we developed a nonparametric regression strategy combining the two phenotypic functions. For this purpose, we performed an additional nonparametric regression of *V*_*i *_values on ${\hat{\pi }}_{ip}$ values using the local linear polynomial estimator as described earlier. In this case, our diagnostic Δ is defined as $1-\sum_{i=1}^{1500}[{\left\{U_{i}-{\hat{\psi }}_{1}({\hat{\pi }}_{ip})\right\}}^{2}+{\left\{V_{i}-{\hat{\psi }}_{2}({\hat{\pi }}_{ip})\right\}}^{2}]/\sum_{\iota =1}^{1500}\{{\left(U_{i}-\bar{U}\right)}^{2}+{\left(V_{i}-\bar{V}\right)}^{2}\}$, where *ψ*_1 _and *ψ*_2 _are the unknown regression functions of ${\hat{\pi }}_{ip}$ corresponding to *U*_*i *_and *V*_*i*_, respectively.

Because the proposed Δ statistic does not consider the direction of the relationship between the squared sib-pair trait difference and the estimated marker IBD scores, there may be concern of an inflated false-positive error rate due to a random negative relationship between the variables under the null hypothesis of no linkage. To circumvent this problem, we ensured that the correlation between the variables is negative for each of the marker positions showing significant evidence of linkage. When we considered the squared differences in conjunction with the squared sums, we additionally verified that the correlation between the squared sums and the estimated IBD score is positive at each of the significant markers.

## Results

We performed our nonparametric regression analyses on all 22 autosomal chromosomes for all 100 available replications. We compared the results of the nonparametric regression with those of the classical Haseman-Elston regression using squared differences [10] and its extension proposed by Elston et al. [7] using mean-corrected cross-products. Because the data involved independent sib pairs, the generalized least squares method of Elston et al. [7] reduced to an ordinary least squares analysis. The RF-IgM levels were corrected for age, sex, and smoking status using linear regression. The IBD computations were performed using the statistical software MERLIN [12]. The nonparametric regressions were performed at all the marker positions separately using the squared sib-pair trait differences only and by combining the squared differences and the squared sib-pair trait sums as discussed in the preceding section. We set a *p*-value threshold of < 0.001 (based on 1000 Monte-Carlo replications under the null hypothesis) to consider a linkage finding to be statistically significant. Since the "answers" were available to us, we considered a linkage peak to be true positive only if both the following criteria were satisfied: it was within a 20 cM window (10 cM on either side) of the true position of a QTL and all other markers within this window provided significant evidence of linkage. Hence, we have assessed the empirical power and the false-positive error rate based on the proportion of replicates yielding significant linkage peaks.

Based on the proposed nonparametric regression, we obtained a linkage peak (with nominal *p*-value < 0.05) at marker STRP11_22 (113 cM) on chromosome 11 in 17 replications using squared differences only and in 31 replications using both the squared differences and the squared sums. All of the other markers within the 20-cM window of the position of the QTL have also provided evidence of linkage at level 0.05 for all these replications. Although given a threshold of *p *< 0.001 for a linkage peak to be statistical significant, the empirical power was only 0.1 when only squared differences were used and 0.23 when both squared differences and squared sums were used, we note that the linkage peak is close to Locus F (115 cM), the QTL controlling RF-IgM. However, the major aim of the study, that is, the belief that the combined use of squared differences and squared sums is more powerful than using only the squared sums is validated by our results. We also found that there was no other marker which provided a statistical evidence of linkage at level 0.05 in more than three replications.

When we used the two linear regression approaches [7,10] for comparing with the nonparametric method, we found that the linkage peak was also at marker STRP11_22 (113 cM) on chromosome 11 for most of the replications both with squared differences as well as mean-corrected cross-products. However, the number of replications giving significant linkage evidence at a nominal level of 0.05 was only 11 for squared differences and 18 for mean-corrected cross-products. When we used a nominal level of 0.001, the corresponding figures were 6 and 13, respectively. Thus, the nonparametric method was more powerful than the linear regression approach both when only squared differences were used as well as when squared sums were combined with squared differences. A summary of the linkage finding on chromosome 11 using the various methods is provided in Table 1.

**Table 1:** Empirical powers at markers near the QTL for RF-IgM on chromosome 11 at level 0.001

Marker	Position	NPD^a^	NPSD^b^	HED^c^	ECP^d^
STRP11_21	110 cM	0.10	0.23	0.06	0.11
STRP11_22	113 cM	0.10	0.23	0.06	0.13
STRP11_23	117 cM	0.10	0.23	0.06	0.13
STRP11_24	124 cM	0.09	0.21	0.05	0.09

^a^NPD, nonparametric regression using squared differences only

^b^NPSD, nonparametric regression using both squared sums and squared differences

^c^HED, Haseman-Elston regression using squared differences [10]

^d^ECP, Elston et al. regression using mean-corrected cross-products [7]

## Conclusion

Our proposed nonparametric method was able to detect linkage near the QTL controlling for RF-IgM level in multiple replicates. The use of the squared sib-pair trait sums in conjunction with the squared differences yielded more power to detect linkage compared to using the squared differences only. We also find that the nonparametric regression, which estimates empirically the nature of local relationship between the phenotypic and genotypic variables, is more powerful than the classical Haseman-Elston regression using squared differences [10] and its extension using mean-corrected cross-products [7], both of which assume a linear relationship between the regression variables. However, for the GAW15 data, the empirical power for the nonparametric regression method at level 0.001 was less than 0.25 even when both the squared differences and sums were used. This may be partially explained by the fact that the RF-IgM levels were simulated under a model with high polygenic and non-shared environmental variances. Moreover, all the sib pairs were affected with rheumatoid arthritis. Thus, the analyses on RF-IgM were performed on a selected sample with reduced variance, resulting in loss of power. However, the fact that the nonparametric method provided more power than the linear regression method seems to suggest that the nonparametric regression is more robust to selected sampling than the linear regression. This is intuitively expected because the nonparametric regression method does not assume any functional form of the relationship between the variables and hence, implicitly uses the selected nature of the sample in estimating the functional relationship. We are currently carrying out extensive simulations under different degrees of selection to evaluate the loss of power of the nonparametric regression under select conditions.

Currently methods use LOD scores as a diagnostic to evaluate the significance of linkage peaks. Because our proposed kernel-smoothing method is nonparametric, a direct comparison with likelihood-based LOD scores is not possible. However, if we consider the *p*-values of our linkage peaks, we can theoretically obtain the LOD scores which would yield these *p*-values. For example, a *p*-value < 0.0001 can be attained for a LOD score greater than 3.29, while a *p*-value < 0.001 can be attained for a LOD score greater than 2.35. We are currently carrying out extensive simulations to compare the performance of the proposed procedure with existing distribution-based methods.

Finally, we emphasize that a major advantage of our method is that it does not assume any probability distribution for RF-IgM levels or any specific functional form of dependence between the regression variables and thus, is robust to violations in underlying model assumptions.

## Competing interests

The author(s) declare that they have no competing interests.
